# Norcantharidin Inhibits Renal Interstitial Fibrosis by Blocking the Tubular Epithelial-Mesenchymal Transition

**DOI:** 10.1371/journal.pone.0066356

**Published:** 2013-06-25

**Authors:** Ying Li, Yan Sun, Fuyou Liu, Lin Sun, Jun Li, Shaobin Duan, Hong Liu, Youming Peng, Li Xiao, Yuping Liu, Yiyun Xi, Yanhua You, Hua Li, Min Wang, Shuai Wang, Tao Hou

**Affiliations:** 1 Division of Nephrology, Second Xiangya Hospital, Central South University, Changsha, P.R. China; 2 Division of Nephrology, The first affiliated hospital, XinJiang Medical University,Uramuq, P.R. China; 3 Department of Clinical Laboratory, Second Xiangya Hospital, Central South University, P.R. China; University of Sao Paulo Medical School, Brazil

## Abstract

Epithelial–mesenchymal transition (EMT) is thought to contribute to the progression of renal tubulointerstitial fibrosis. Norcantharidin (NCTD) is a promising agent for inhibiting renal interstitial fibrosis. However, the molecular mechanisms of NCTD are unclear. In this study, a unilateral ureteral obstruction (UUO) rat model was established and treated with intraperitoneal NCTD (0.1 mg/kg/day). The UUO rats treated with NCTD showed a reduction in obstruction-induced upregulation of α-SMA and downregulation of E-cadherin in the rat kidney (P<0.05). Human renal proximal tubule cell lines (HK-2) stimulated with TGF-β_1_ were treated with different concentrations of NCTD. HK-2 cells stimulated by TGF-β_1_ in vitro led to downregulation of E-cadherin and increased de novo expression of α-SMA; co-treatment with NCTD attenuated all of these changes (P<0.05). NCTD reduced TGF-β_1_-induced expression and phosphorylation of Smad2/3 and downregulated the expression of Snail1 (P<0.05). These results suggest that NCTD antagonizes tubular EMT by inhibiting the Smad pathway. NCTD may play a critical role in preserving the normal epithelial phenotype and modulating tubular EMT.

## Introduction

Norcantharidin (NCTD), an important derivative of cantharidin, is a specific protein phosphatase inhibitor with anti-cancer, inflammation modulation, anti-fibrosis, anti-oxidation and leukocytic effects [1]. Recently, our research showed that NCTD significantly alleviated renal interstitial fibrosis in rat models of protein overload nephropathy [2] and diabetic nephropathy [3]. NCTD inhibited the proliferation and expression of fibronectin (FN) in renal tubular epithelial cells stimulated by albumin in vitro [4] and decreased the expression of extracellular matrix (ECM) and TGF-β_1_ in HK-2 cells stimulated with high glucose [5]. As a result, NCTD is considered a promising antifibrotic drug [6]. However, the molecular mechanisms by which NCTD contributes to the inhibition of tubulointerstitial fibrosis are unclear

Renal interstitial fibrosis is the final common pathway through which chronic kidney diseases progress to end-stage renal failure [7]. The process of renal interstitial fibrosis includes tubular atrophy, myofibroblast accumulation and ECM deposition. Epithelial-mesenchymal transition (EMT) is an important pathway in myofibroblast production and is a key mechanism in the pathogenesis and progression of renal interstitial fibrosis [8]–[10]. In EMT, the loss of epithelial cell adhesion molecules, such as epithelial (E)-cadherin, are replaced by the mesenchymal marker alpha-smooth muscle actin (α-SMA)[10], [14]. However, the role of EMT in renal interstitial fibrosis in vivo has recently been challenged by two different groups [11], [12], which conflicts with previous conclusions of Iwano et al [13]. Using cell lineage tracking techniques to track renal epithelial cells, the authors of those two groups found no evidence to suggest that epithelial cells migrate outside of the tubular basement membrane and differentiate into interstitial myofibroblasts in vivo. This conflicting results may be due to technical differences, underlining the complexity of the EMT process. The degree to which the EMT process contributes to kidney fibrosis remains a matter of intense debate and is likely to be context-dependent or an adaptive response to a hostile environment, chronic stress or injury. Of the numerous cytokines that regulate EMT, TGF-β_1_ is a key mediator that promotes myofibroblast development by inducing expression of the α-SMA phenotype. TGF-β_1_ is considered a key mediator in renal fibrosis and induces renal scarring by activating the downstream Smad signaling pathway [15]. Smad2 and Smad3 are critical downstream mediators responsible for the biological effects of TGF-β_1_. Snail1 is a key transcription factor that promotes EMT, fibroblast migration and renal fibrosis [16], [17]. Manipulating downstream TGF-β_1_ signaling represents a viable therapeutic target for reversing EMT and renal interstitial fibrosis. We hypothesized that the effects of NCTD in ameliorating renal interstitial fibrosis may be related to inhibition or reversal of tubular EMT.

In this study, we examined the effects of NCTD on renal tubular EMT in a rat model with unilateral ureteral obstruction (UUO) and HK-2 cells. We explored the mechanisms by which NCTD inhibits renal tubular EMT and renal interstitial fibrosis.

Our results suggest that NCTD may play a critical role in preserving the normal epithelial phenotype and modulating tubular EMT by inhibiting the TGF-β_1_/Smad pathway.

## Materials and Methods

### Ethics statement

All experiments were performed in accordance with the animal experimental guidelines issued by the Animal Care and Use Committee at Xiangya Medical School of Central South University. This study was approved by the Animal Care and Use Committee of the 2nd Xiangya Hospital (protocol approval number 2008-S 062).

### Animal model

Healthy 8-week-old male Sprague-Dawley rats weighing 200–220 g (SPF, certification number×2003115403) were purchased from the Animal Department at Xiangya School of Medicine, Central South University. At harvest, each rat was anesthetized by intraperitoneal injection with 10% chloralic hydras. A unilateral ureteral obstruction (UUO) model was established by surgically opening the dorsal surface of the rat with a left lateral incision. The left ureter was ligated with 4–0 silk sutures at two points and severed between the ligatures to prevent a retrograde urinary tract infection after routine skin preparation and sterilization. The incision was closed in layers. Sham-operated rats were subjected to the same anesthesia, incision and closure, but the left ureter was manipulated without ligation [18]. The total time spent in surgery was 10–15 min per rat. Respiratory rhythm and frequency were monitored during the procedure. UUO rats were treated with intraperitoneal NCTD (0.1 mg/kg/day) (n = 6). The rats in the sham group and UUO rats that were not treated with NCTD were administered with equal volumes of sterile saline (n = 6 in each group). The rats were sacrificed 14 days after surgery by exsanguination through cardiac puncture under general anesthesia. The abdomen of the rat was opened and both kidneys were removed into ice-cold saline. Whole blood samples were obtained from the abdominal aorta of each rat for serum creatinine measurement.

### Cell culture

HK-2 cells from American Type Culture Collection (ATCC) were grown in DMEM supplemented with 10% FBS at 37°C in humidified 5% CO_2_ in air. The cells were subcultured at 80% confluence using 0.05% trypsin with 0.02% EDTA. The cells were diluted to 1×10^6^ cells/ml by mixing with medium and transferred to 6-well plates. The experiment was performed on cells at 80% confluence. The HK-2 cells were divided into the following five treatment groups: control, TGF-β_1_ 5 ng/ml + NCTD 0 μg/ml, TGF-β_1_ 5 ng/ml + NCTD 0.5 μg/ml, TGF-β_1_ 5 ng/ml + NCTD 1 μg/ml and TGF-β_1_ 5 ng/ml + NCTD 2.5 μg/ml.

### Immunohistochemistry staining

Paraffin-embedded whole kidney tissue sections were dewaxed, hydrated, treated with 5 mmol/L levamisole for blocking endogenous alkaline phosphatase and incubated with blocking serum for 30 min at room temperature to reduce nonspecific background staining. Sections were rehydrated in PBS with 0.1% BSA for 15 min before the appropriate blocking serum was added for an additional 15 min. To detect the expression of α-SMA, E-cadherin and TGF-β_1_ proteins in the kidneys, sections (4 µm thick) were incubated with rabbit anti-rat α-SMA polyclonal antibody (1∶50, Abcam, Inc., London, UK), E-cadherin (1∶50, Santa Cruz, Inc., CA, USA) and TGF-β1 (1∶25, Santa Cruz, Inc., CA, USA). After rinsing, the sections were incubated with HRP-labeled biotinylated goat anti-rabbit IgG at 37°C for 30 min (Abcam, Inc., London, UK) and processed using an alkaline phosphatase-streptavidin-biotin immunoperoxidase method (Maixin Biotechnological Company, Shenzhen, China). The sections incubated with non-immune rabbit serum rather than the primary antibodies were used as negative controls. Nuclei were counterstained slightly with hematoxylin. All images were semi-quantitatively analyzed using the computer- assisted image-Pro Plus software, version 6.0 (Media Cybernetics, Bethesda, MD, USA). To quantitate the the positive expression area of α-SMA, E-cadherin and TGF-β_1_ in the tubulointerstitial compartment, 50 fields consecutively selected in the cortical areas of the kidney were examined at a magnification of ×100. Fields containing glomerular and large arteries were excluded. The analysis was made by the authors without knowledge about clinical data. Values were expressed as the average optical density (AOD), which is defined as the ratio of the positive target optical density and the positive area percentage (IOD/Area).

### Immunofluorescence staining

Cells grown on coverslips in 6-well dishes were fixed in 4% paraformaldehyde in phosphate buffered saline (PBS) for 30 min at room temperature, followed by permeabilization with 0.1% TritonX-100 in PBS for 5 min. The cells were rinsed in PBS (pH 7.4) three times for 5 min. After blocking in 5% BSA and 1% TritonX-100/PBS for 30 min at room temperature, the cells on the coverslips were incubated with primary rabbit anti-human α-SMA polyclonal antibody (1∶100, Abcam, Inc., London, UK) and mouse anti-human E-cadherin monoclonal antibody (1∶100, Santa Cruz, Inc., California, USA) for 1 h at room temperature. After rinsing with PBS three times for 10 min with agitation, the cells were incubated with AlexaFluora 568 (red) or AlexaFluora 488 (green) conjugated secondary antibodies (Molecular Probes, Eugene, OR, USA) for 30 min at room temperature. The nuclei were counterstained with 4′,6′-diamidino-2-phenylindole dihydrochloride. All of the images were semi-quantitatively analyzed, and the slides were mounted and viewed using a fluorescent microscope. For analysis of α-SMA and E-cadherin expression, fluorescent intensity was quantified by measuring intensity in the cells using Image-Pro Plus software, version 6.0 (Media Cybernetics, Bethesda, MD, USA). Data were analyzed from three sections of one sample, and there were six samples for each group.

### Western blot Analysis

The whole kidney tissues and HK-2 cells were lysed in RIPA lysis buffer and protease inhibitor PMSF at 4°C. The lysate was clarified by centrifugation at 12,000 rpm for 30 min at 4°C, and the supernatant was used for the experiments. Nuclear protein was extracted using a nuclear and cytoplasmic protein extraction kit according to the manufacturer's instructions. The protein concentration was determined using the Bradford method. To denature the extracted proteins and expose the domain typically recognized by antibodies, a loading buffer with the anionic denaturing detergent sodium dodecyl sulfate (SDS) was used. The mixture was boiled at 95°C for 5 min. Protein was loaded on a SDS polyacrylamide gel for electrophoresis and transferred to PVDF membrane using the transfer buffer. The membrane was incubated for 2 h at room temperature under agitation with 2% BSA to prevent nonspecific background binding. The immunoblots from the rat kidney were incubated overnight at 4°C with primary antibodies, including rabbit anti-rat α-SMA, E-cadherin and TGF-**β**_1_ (1∶3000). In the immunoblots from the HK-2 cells, E-cadherin antibody (1∶3000), α-SMA antibody (1∶4000), rabbit anti-human Smad2/3 polyclonal antibody (1∶2000, Cell Signaling, Inc., Danvers, MA, USA) and P-Smad2/3 polyclonal antibody (1∶2000, Santa Cruz, Inc., CA, USA) and rat anti-human Snail 1 polyclonal antibody (1∶1500, Cell Signaling, Inc., Danvers, MA, USA) were used. After several washes, the membranes were incubated with agitation for 1 h at room temperature with goat anti-rabbit horseradish peroxidase-conjugated secondary antibody. Chemiluminescence was used to detect specific protein bands using ECL detection kits, and the results were recorded on radiograph film. Optimally exposed autoradiographs were digitally scanned and analyzed using a Kodak high-sensitivity imaging system (Carestream Health, Inc, USA). The intensity of the identified bands was quantified by densitometry. Results were normalized to β-actin and expressed as arbitrary densitometry units.

### RT-PCR

Trizol RNA extraction was used to extract the total RNA from the renal tissue. The total RNA, weighing 2.5 μg, was used for cDNA synthesis according to the instructions in the Revert Aid First Strand cDNA Synthesis Kit (Fermentas). Gene sequences were verified in Genebank, and primers were designed according to primer design principles using Primer 5 software. The primer sequences are shown in Table 1. PCR amplification of the various products was performed under the following reaction conditions: a) α-SMA mRNA: pre-degeneration at 95°C for 5 min, degeneration at 94°C for 45 sec, annealing at 56°C for 45 sec and extension at 72°C for 45 sec, with 30 cycles total and a final extension at 72°C extension for 10 seconds terminated at 4°C; b) E-cadherin mRNA: pre-degeneration at 95°C for 3 min, degeneration at 94°C for 30 sec, annealing at 60°C for 30 sec and extension at 72°C for 30 sec, with 34 cycles in total and a final extension at 72°C for 10 sec terminated at 4°C; for 30 sec, annealing at 58°C for 30 sec and extension at 72°C for 30 sec, with 30 cycles total and a final extension at 72°C extension for 10 seconds terminated at 4°C; and d) β-actin mRNA: pre-degeneration at 95°C for 5 min, degeneration at 94°C for 30 sec, annealing at 58°C for 30 sec and extension at 72°C for 30 sec, with 25 cycles total and a final extension at 72°C extension for 7 seconds terminated at 4°C.

**Table 1:** RT-PCR primers for the rat kidney.

Gene	Upstream sequence	Downstream sequence
**α-SMA**	5'-TCC TGA CCC TGA AGT ATC CG-3'	5'-TCT CCA GAG TCC AGCA CAA T-3'
**E-cadherin**	5'-GTC AAA CGG CAT CTA AAG C-3'	5'-CAA AGA CCT CCT GGA TAA ACT-3'
**TGF-β1**	5'-CCG CAA CAA CGC AAT C-3'	5'-ATG AGG AGC AGG AAG GGT -3'
**β-actin**	5'-ATG AGG AGC AGG AAG GGT -3'	5'-ACCCAGGAAGGAAGGCT -3'

In HK-2 cells, E-cadherin, α-SMA, and Snail 1 mRNA expression were detected by RT-PCR. PCR was followed by 40 cycles of 10 sec at 95°C, 60 sec at 60°C, 60 sec at 72°C and one cycle of 10 min at 72°C. The housekeeping gene β-actin served as a reference and was co-amplified with E-cadherin, α-SMA, and Snail 1. The primer sequences for the HK-2 cells are shown in Table 2. All of the oligonucleotide primers were designed by the Ying Jun Limited Company (Shanghai, China). After PCR amplification, 8 µl of each amplification product was electrophoresed on a 2% agarose gel at 100 V for 40 min, then the agarose gel was visualized and transillumination under UV light. The images were obtained using a gel imaging system for pictures and Labwork 4.0 photography analysis software (Gene Company Limited, Hongkong, China) to quantify gray value of the bands observed. Light absorbance values were determined using gel image analysis software, and the semi-quantitative value was presented as a ratio between the light absorbance of each index and the light absorbance of β-actin. SYBR green, a fluorescent dye that only binds to double-stranded DNA, was used as the fluorescent probe. Fluorescence was emitted proportional to the amount of cDNA amplified by RT-PCR.

**Table 2:** RT-PCR primers for the HK-2 cells.

Gene	Upstream sequence	Downstream sequence
**α-SMA**	5'CTG TTC CAG CCA TCC TTC ATC 3'	5' GCT GGC TCA AGT CAA AGT CC 3'
**E-cadherin**	5'TTG CAA ATT CCT GCC ATT C 3'	5' GCT GGC TCA AGT CAA AGT CC 3'
**Smad2**	5' TTGCTGAGTGCCTAAGTGAT 3'	5'ACAGACTGAGCCAGAGAGC 3'
**Smad3**	5'GGCTTTGAGGCTGTCTACCA 3'	5'CATCTGGGTGAGGACCTTGT 3'
**Snail 1**	5'GAA AGG CCT TCA ACT GCA AA 3'	5' GTA CTT GCG CTC AGG AGG AG 3'
**β-actin**	5' ACTCTTCCAGCCTTCCTTCC3'	5' GTACTTGCGCTCAGGAGGAG 3'

### Statistical methods

SPSS 13.0 software was used for statistical analysis. The results are presented as Means ± SD. Measurement data were analyzed using one-way analysis of variance (ANOVA), and the LSD t-test was used for multiple comparisons of two sample means. P<0.05 was considered statistically significant.

## Results

### NCTD regulates the expression of α-SMA, E-cadherin and TGF-β_1_ in the kidneys of UUO rats

**Figure 1:**
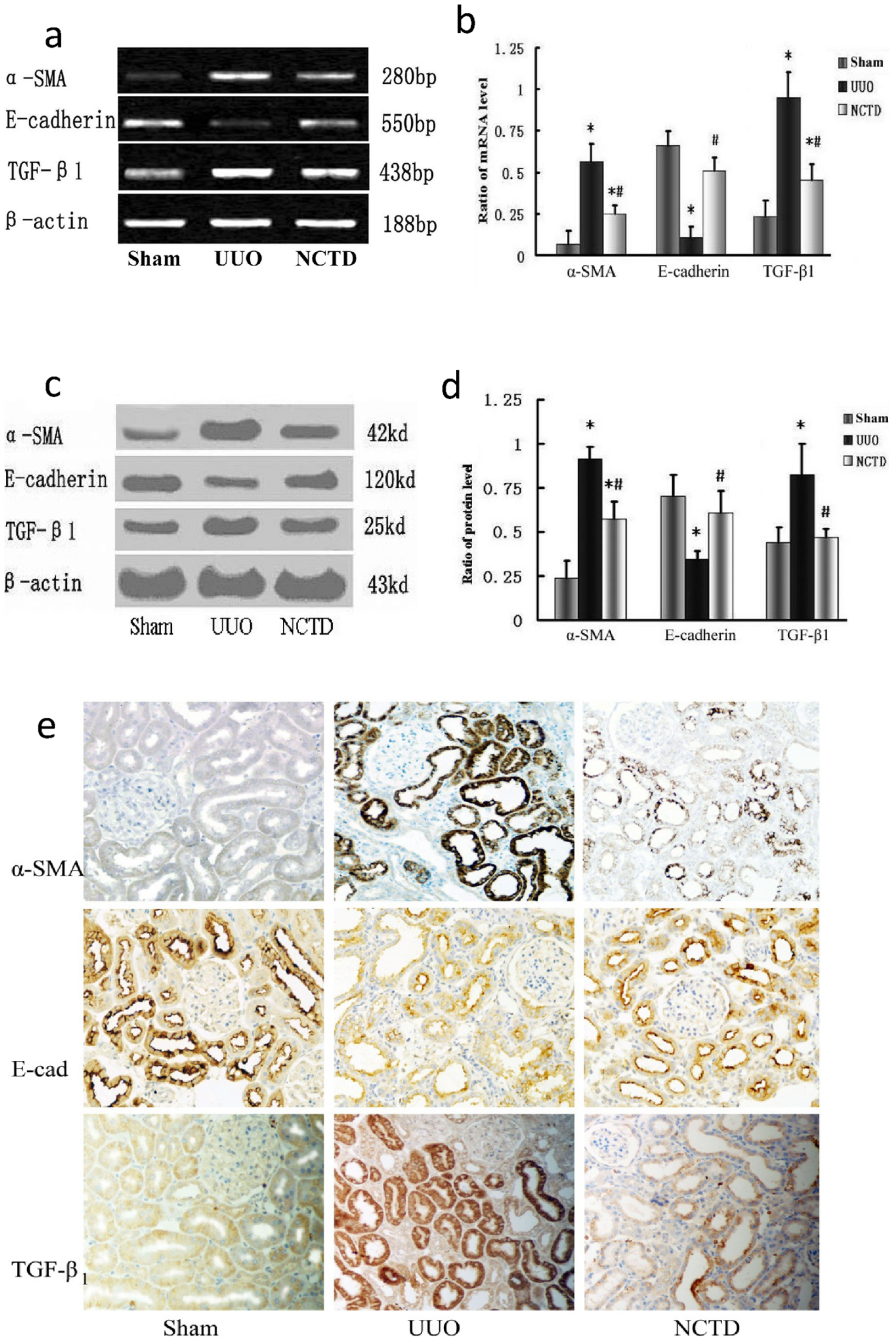
NCTD regulates the expression of α-SMA, E-cadherin and TGF-β1 in a rat UUO model. (a and b) Representative RT-PCR results (a) and graphic presentation (b) show α-SMA, E-cadherin and TGF-β1 mRNA in different treatment groups.* P<0.05 vs. sham group and #P<0.05 vs. UUO group. (c and d) Representative Western blot (c) and quantitative data (d) for α-SMA, E-cadherin and TGF-β1 protein expression in various groups. * P<0.05 vs. sham group and #P<0.05 vs. UUO group. (e) Immunohistochemical staining shows α-SMA, E-cadherin and TGF-β1 protein expression in the rat kidney of different treatment groups (×200).

We investigated the effects of NCTD on the expression of α-SMA, E-cadherin and TGF-β_1_ in obstructive nephropathy, a well characterized and widely used model of renal interstitial fibrosis**.** The expression of α-SMA and TGF-β_1_ mRNA in the UUO group was significantly increased, while the expression of E-cadherin mRNA decreased (*P*<0.05). However, downregulation of α-SMA and TGF-β_1_ mRNA and upregulation of E-cadherin mRNA were observed in the NCTD group compared with the UUO group. The difference between the two groups was significant (*P*<0.05, Fig. 1a and b). Similarly, α-SMA and TGF-β_1_ protein expression in the obstructed kidney significantly increased and E-cadherin protein decreased based on Western blot analyses of whole kidney lysates (*P*<0.05). α-SMA and TGF-β_1_ protein expression markedly decreased with NCTD treatment, while E-cadherin protein increased. All the differences between these three groups had statistical significance (*P*<0.05, Fig. 1c and d). Immunohistochemistry staining showed that α-SMA protein expression in the renal tubular epithelial cells and interstitium was significantly elevated in the rat kidney after obstructive injury, while expression of the E-cadherin protein decreased. TGF-β_1_ protein expression in renal tubular epithelial cells, stromal cells and infiltrated inflammatory cells also increased. NCTD markedly reduced the changes in these markers (Fig. 1e). These data collectively indicate that NCTD is a potent agent for reversing renal tubular EMT in UUO rats.

### TGF-β_1_ increases α-SMA expression in HK-2 cells

**Figure 2:**
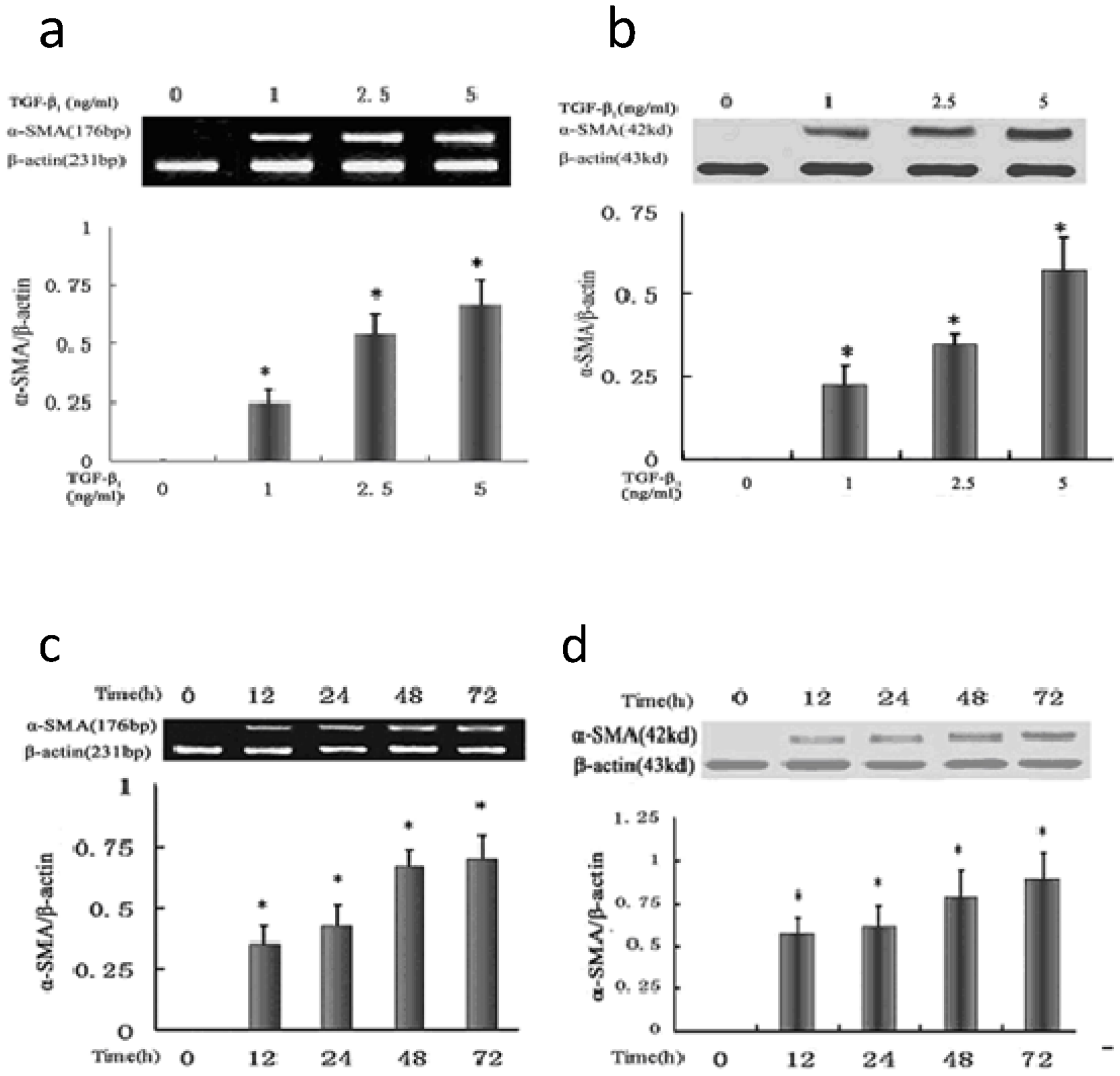
TGF-β1 induces the expression of α-SMA in HK-2 cells. (a and b) Representative RT-PCR (a) and Western blot results (b) show that TGF-β1 induced expression of α-SMA mRNA and protein in HK-2 cells in a dose-dependent manner. * P<0.05 vs. negative control group (TGF-β1 0 ng/ml). (c and d) Representative RT-PCR (c) and Western blot results (d) show that TGF-β1 induced expression of α-SMA mRNA and protein in HK-2 cells in a time-dependent manner. * P<0.05 vs. negative control group (TGF-β1 0 ng/ml).

We examined the suitable dose and length of exposure of TGF-β_1_ on α-SMA expression in HK-2 cells. α-SMA expression in HK-2 cells following treatment with different concentrations of TGF-β_1_ (0, 1.25, 2.5 and 5 ng/ml) for 72 h was analyzed. RT-PCR and Western blot analysis showed that the mRNA and protein levels of α-SMA were upregulated, and a dose-dependent increase was observed (*P*<0.05 between each concentration point) (Fig. 2a and b). α-SMA expression in HK-2 cells after TGF-β_1_ treatment after different periods of time was evaluated. α-SMA mRNA and protein expression in HK-2 cells were detected at 0 h, 12 h, 24 h, 48 h and 72 h after treatment with 5 ng/ml TGF-β_1_. There was a gradual time-dependent increase in α-SMA mRNA and protein expression (*P*<0.05 between each time point). (Fig. 2c and d). According to the data, HK-2 cells treated with 5 ng/ml TGF-β_1_ for 48 h were used in subsequent experiments.

### NCTD regulates the expression of α-SMA and E-cadherin in HK-2 cells stimulated by TGF-β_1_

**Figure 3:**
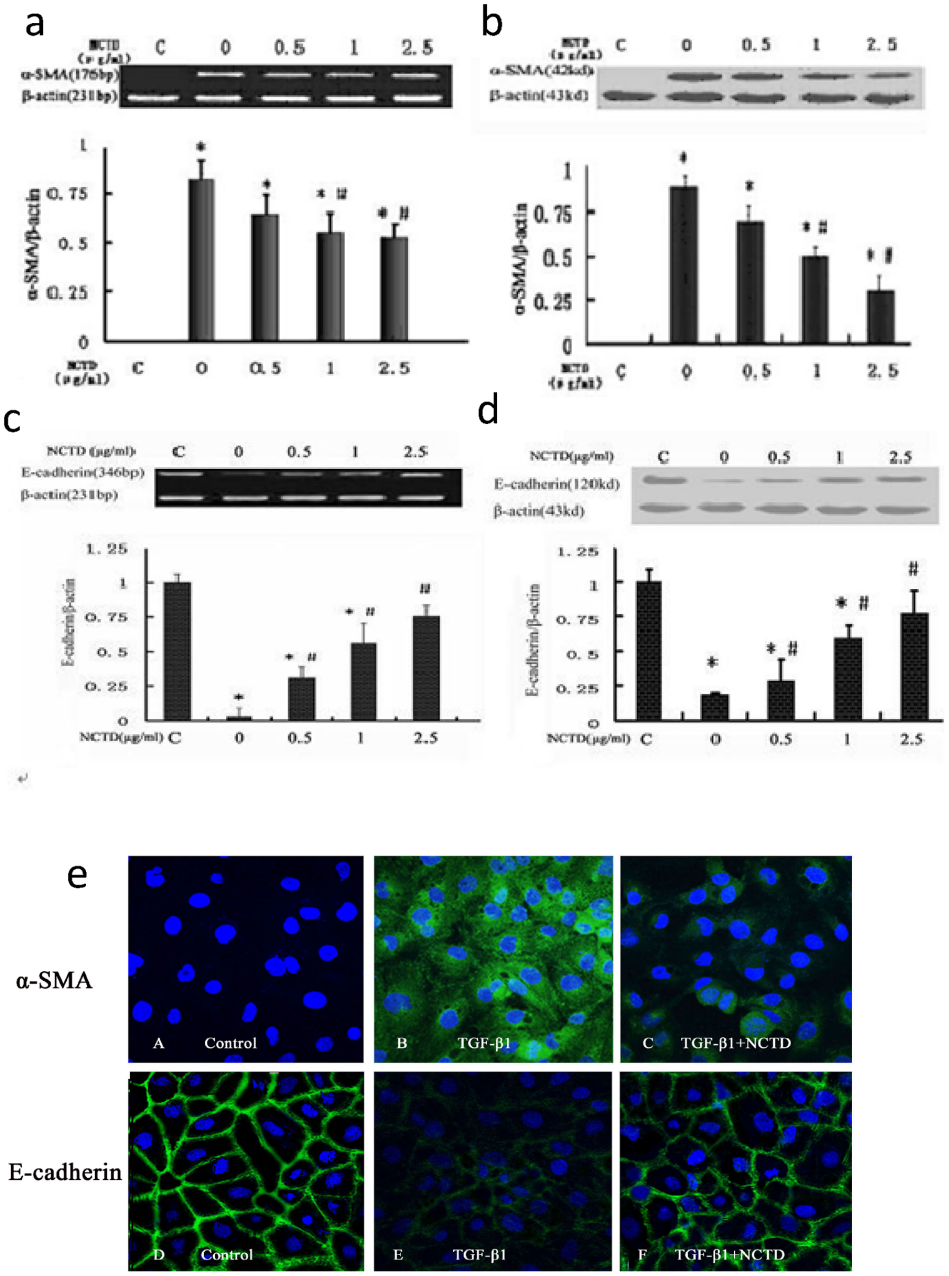
NCTD regulates expression of α-SMA and E-cadherin in HK-2 cells stimulated by TGF-β1. (a and b) Representative RT-PCR (a) and Western blot (b) results show that NCTD inhibited the expression of α-SMA mRNA and protein in HK-2 cells stimulated by TGF-β1. * P<0.05 vs. negative control group (TGF-β1 0 ng/ml), #P<0.05 vs. positive control group (TGF-β1 5 ng/ml + NCTD 0 µg/ml). (c and d) NCTD increased expression of E-cadherin mRNA and protein in HK-2 cells stimulated by TGF-β1.* P<0.05 vs. negative control group (TGF-β1 0 ng/ml) and #P<0.05 vs. positive control group (TGF-β1 5 ng/ml + NCTD 0 µg/ml). (e) NCTD (2.5 μg/ml) regulated the phenotypic changes in HK-2 cells stimulated with TGF-β1 (Immunofluorescence×200).

After HK-2 cells were treated with 5 ng/ml TGF-β_1_ for 48 h, there was a marked increase in α-SMA expression and a decrease in E-cadherin expression. Different concentrations of NCTD for 48 h significantly prevented above changes of the α-SMA and E-cadherin expression in HK-2 cells. NCTD prevented the increased levels of α-SMA and the reduced E-cadherin levels in a dose-dependent manner, with the highest concentration significantly inhibiting the changes. All of these results were confirmed by RT-PCR and Western blot, which showed reduced expression of α-SMA and increased expression of E-cadherin after treatment with NCTD compared to treatment with TGF-β_1_ without NCTD intervention (Fig. 3a–d). Immunofluorescence results showed that α-SMA protein expression was increased in the cytoplasm and E-cadherin protein expression was decreased in the cytomembrane of HK-2 cells stimulated with TGF-β_1_. However, NCTD treatment obviously downregulated α-SMA protein expression and upregulated E-cadherin protein expression (Fig. 3e). These results indicate that NCTD prevented de novo expression of the myofibroblast marker α-SMA in HK-2 cells and loss of the epithelial marker E-cadherin.

### NCTD inhibits the expression and phosphorylation of Smad2 and Smad3 in HK-2 cells stimulated by TGF-β_1_

**Figure 4:**
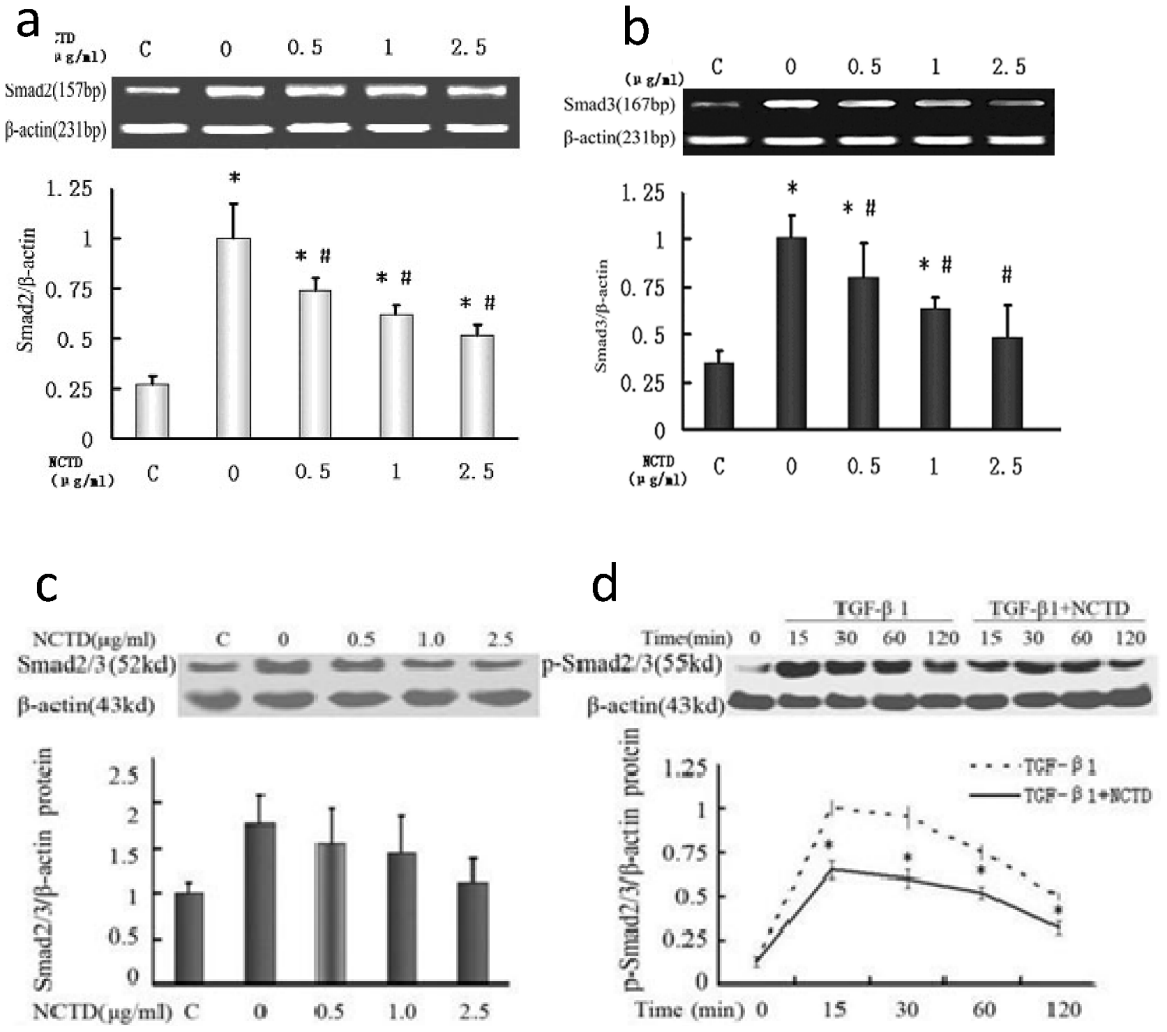
NCTD inhibits expression of Smad2 and Smad3 in HK-2 cells induced by TGF-β1. (a and b) NCTD inhibited expression of Smad2 and Smad3 mRNA in HK-2 cells induced by TGF-β1. * P<0.05 vs. negative control group (TGF-β1 0 ng/ml) and # P<0.05 vs. positive control (TGF-β1 5 ng/ml + NCTD 0 µg/ml). (c and d) NCTD reduced expression of Smad2/3 and p-Smad2/3 proteins in HK-2 cells induced by TGF-β1. * P<0.05 vs. negative control group (TGF-β1 0 ng/ml) and # P<0.05 vs. positive control group (TGF-β1 5 ng/ml + NCTD 0 µg/ml).

This study showed that the expression of Smad2 and Smad3 mRNA was upregulated in HK-2 cells stimulated by TGF-β_1_ compared with the control group. However, NCTD significantly decreased the expression of Smad2 and Smad3 in a dose-dependent manner (Fig. 4a and b). Western blot indicated that the expression level of the Smad2/3 protein in HK-2 cells stimulated by TGF-β_1_ was significantly higher than the expression in control group. The NCTD intervention dose downregulated Smad2/3 protein expression (Fig. 4c). The pSmad2/3 protein in HK-2 cells stimulated by TGF-β1 increased during the first 15 minutes of exposure, then gradually declined after prolonged exposure to TGF-β_1_ stimulation. NCTD downregulated the pSmad2/3 protein after 15, 30, 60 and 120 min (*P*<0.05 between each time point) (Fig. 4d). These data show that NCTD is effective in inhibiting the expression and phosphorylation of Smad2/3 protein in HK-2 cells.

### NCTD reduces the expression of Snail 1 in HK-2 cells stimulated by TGF-β1

**Figure 5:**
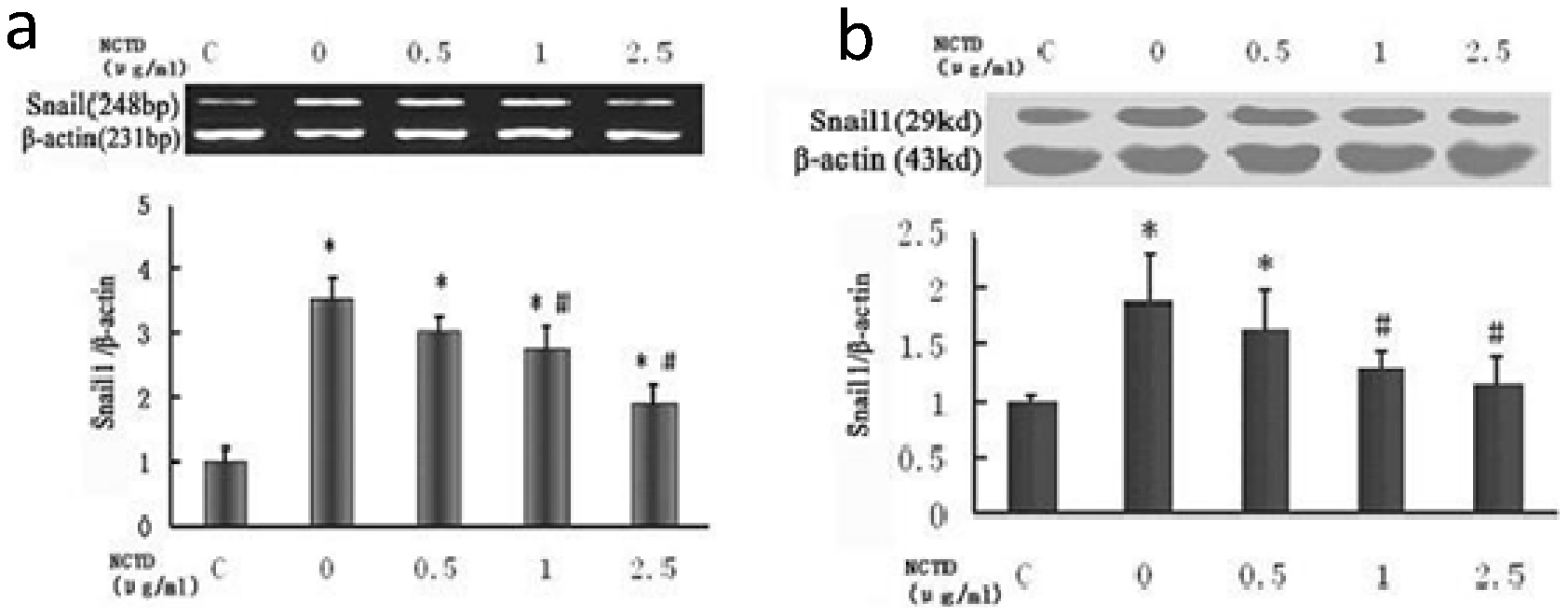
NCTD reduces the expression of Snail 1 in HK-2 cells stimulated by TGF-β1. (a) NCTD reduced expression of Snail 1 mRNA in HK-2 cells stimulated by TGF-β1. (b) NCTD reduced expression of Snail 1 protein in HK-2 cells stimulated by TGF-β1. * P<0.05 vs. negative control group (TGF-β1 0 ng/ml) and #P<0.05 vs. positive control group (TGF-β1 5 ng/ml + NCTD 0 µg/ml).

This study showed that all concentrations of NCTD significantly reduced Snail 1 expression in HK-2 cells after TGF-β_1_ treatment for 48 h compared with the control group, which was confirmed by RT-PCR and Western blot. However, the concentration of NCTD was inversely correlated with incremental effects on Snail 1 expression (Fig. 5a and 5b).

## Discussion

Interstitial fibrosis is a common pathway of progressive renal disease, leading to end-stage renal failure regardless of the etiology. Interstitial fibrosis is the strongest morphological predictor of clinical outcome and progression [19]. Fibroblasts in the renal interstitium are considered the principal source of the fibrillar matrix, and tubulointerstitial fibrosis is inevitably associated with a robust accumulation of fibroblasts [20]. *In vivo* studies have indicated that the majority of fibroblasts in the damaged kidney could originate from the tubular epithelium through the process of tubular EMT [21]. Renal tubular EMT is an important event in the pathogenesis of tubulointerstitial fibrosis and is characterized by loss of epithelial features and acquisition of mesenchymal markers under excessive exposure to various profibrotic cytokines [22]. Thus, inhibiting myofibroblast accumulation is critical in preventing tubulointerstitial fibrosis and preserving renal function.

TGF-β_1_ is a strong profibrotic cytokine that activates the expression of ECM components, such as collagens I and IV and fibronectin, and inhibits collagen-degrading enzymes, such as certain matrix metalloproteinases (MMPs) involved in controlling ECM homeostasis. This leads to excessive ECM accumulation and fibrosis. In addition, TGF-β_1_ is a well-known fibrogenic cytokine in renal disease and plays a key role in EMT. The functional role of TGF-β_1_ in EMT and renal fibrosis is demonstrated by blocking TGF-β_1_ with neutralizing TGF-β_1_ antibodies and antisense oligonucleotides to prevent or ameliorate renal fibrosis *in vivo and in vitro* [23], [24]. The downstream signaling of TGF-β_1_ is profoundly related to a family of Smad proteins that stimulate fibrosis (Smad2 and Smad3) or inhibit fibrosis (Smad7). a-SMA -positive myofibroblasts were identified as the primary cell type responsible for interstitial matrix accumulation in fibrotic diseases, including in the kidney. Moreover, an α-SMA phenotype is considered as a useful marker for myofibroblast differentiation in tubulointerstitial fibrosis. EMT is an orchestrated, highly regulated process that consists of loss of epithelial cell adhesion, which is characterized by the loss E-cadherin expression and increased expression of a-SMA. In this study, TGF-β_1_ caused enhancement of a-SMA expression in dose and time-dependent manners. This supports the suggestion that TGF-β_1_ can induce transdifferentiation of HK-2 cells into myofibroblasts. However, few therapeutic options are currently available. There is an urgent need to discover new therapeutic agents, particularly specific antagonists targeting TGF-β_1_ and multiple injurious pathways, to more effectively arrest or even reverse renal tubulointerstitial fibrosis.

NCTD, an inhibitor of protein phosphatases, including PP1 and PP2A, is the demethylated analog of cantharidin. It also possesses anticancer activity [6]. Cantharidin can inhibit migration, invasion and adhesion of highly metastatic human ovarian carcinoma cell lines by downregulating expression of the NF-kB P65 subunit and vascular endothelial growth factor (VEGF). It has been reported that NCTD decreased the ratio of MMP2 to TIMP2 and reduced cellular mortality, exerting anti-invasive activity in human gallbladder carcinoma cells. The anti-metastatic activity of NCTD correlates with multiple factors involved in the EMT process. Our previous findings showed that NCTD exhibited a protective function in tubular interstitial fibrosis [2]–[6], during which EMT plays an important role. According to our preliminary studies, NCTD is beneficial in renal tubulointerstitial fibrosis, although its underlying pharmacological mechanisms are not well understood. We speculate that NCTD may ameliorate renal fibrosis by inhibiting the EMT process.

The experimental UUO rat model is widely used to study progressive renal fibrosis. The obstructed kidney after UUO exhibits significant interstitial inflammatory cell infiltration and tubulointerstitial fibrosis [25]. Matrix-producing fibroblasts are thought to convert into myofibroblasts characterized by de novo activation of α-SMA during fibrogenesis. Fibroblasts in the kidney may be derived from resident interstitial cells, mesenchymal and/or hematopoietic stem cells, periadventitial cells or the EMT process [21]. In the UUO model of renal fibrosis, up to 36% of the cells that produce the extracellular matrix are derived from tubular epithelial cells by EMT [13]. In this study, we used a UUO rat model and HK-2 cells induced by TGF-β1 to determine the effects of NCTD on tubular EMT. The results show that NCTD decreased upregulation of α-SMA and TGF-β_1_, while downregulation of E-cadherin was decreased in the rat kidney. NCTD also reduced α-SMA expression in vitro and increased E-cadherin expression in HK-2 cells induced by TGF-β_1_. However, EMT is presently a controversial subject and its existence has been recently challenged. Confirmatory studies on epithelial cells are an important source of myofibroblasts through EMT in vivo are lacking [11], [12]. Our in vitro study suggests that NCTD may act by inhibiting expression of TGF-β_1_, thereby delaying or preventing TGF-β_1_ mediated EMT and renal interstitial fibrosis, but this does not constitute direct proof that this actually happens in vivo. It need to be explored in the future study to confirm the inhibitory effect of NCTD on tubular EMT.

TGF-β signaling pathways are activated by a short phosphorylation cascade, from receptor phosphorylation to subsequent phosphorylation and activation of downstream signal transducer R-Smads (receptor-activated Smads). Upon ligand stimulation, R-Smad proteins are phosphorylated in the SXS motif by TGF-β type I receptors, leading to the activation of a series of downstream events. The results of our in vitro experiment showed a increased expression of Smad2/3 protein and phosphorylation stimulated by TGF-β_1_, which was reduced by co-treatment with NCTD, providing an evidence that NCTD has an inhibitory effect on TGF-β_1_/Smad pathway. Snail 1, a zinc finger transcription factor, has been characterized as a key regulator of EMT. Many studies have shown that Snail 1 binds to specific DNA sequences called E-boxes in the promoter of the E-cadherin gene to repress transcription of E-cadherin [26], [27]. E-cadherin is a major gene in the epithelium, which is an important determinant in maintaining the epithelial phenotype [28]–[31]. EMT was rapidly induced within 24 hours after UUO, and the Snail1 transcription factor was upregulated, preceding the induction of α-SMA [32]. Although activity of the Snail 1 gene is required during embryonic development to form different tissues and organs, the gene is repressed in adults to maintain epithelial integrity and homeostasis [33]. Pathological activation of Snail 1 in adult tubular epithelial cells is sufficient to induce tubulointerstitial fibrosis. Snail 1 mRNA has been shown to specifically localized to renal tubular epithelial cells in wild-type mice 7 days after UUO [34]–[36]. A study recently reported that overexpression of Snail 1 in the epithelial nucleus contributes to EMT. Snail 1 mRNA and protein were upregulated in the tubular cells of rat kidneys in the UUO model and human proximal tubule cells treated with TGF-β_1_, demonstrating that Snail 1 is involved in tubular EMT [37]. However, whether NCTD can regulate the expression of Snail 1 is unclear. Our experiment observed the expression of Snail 1 mRNA and protein in HK-2 cells induced by TGF-β_1_. After NCTD intervention for 48 h, Snail 1 expression was significantly downregulated, suggesting an inhibitory effect of NCTD on TGF-β_1_-induced Snail 1 expression in HK-2 cells. We speculate that NCTD can inhibit tubular EMT in TGF-β_1_-induced HK-2 cells, which may contribute to inhibition of Snail 1. However, we only find NCTD is capable of stopping the existence of events that occur downstream to binding of TGF-β_1_ to its receptor by blocking Smad and Snail 1 expression in vitro. All of these findings also need to be proved that NCTD exerts the same effect *in vivo*.

It is well known that the phosphorylation cascade from receptor to Smad proteins plays an important role in the activation of TGF-β signaling. Dephosphorylation of receptors and Smad proteins also contributes to the duration and intensity of TGF-β signaling. This reversible phosphorylation provides a balance for proper functioning of signaling molecules. PP2A is a well-known protein phosphatase that associates with TGF-β receptors [38]. The regulatory function of PP2A in regulating TGF-β signaling has been demonstrated. PP2A may dephosphorylate Smad3 under hypoxic conditions [39]. NCTD has a remarkable inhibitory effect on protein phosphatases, including PP1 and PP2A. What is more important, NCTD has a stronger inhibitory effect on PP2A compared to PP1 (IC50  = 2.69 mM vs. 10.3 mM) [40]. However, whether NCTD plays an antifibrotic role by inhibiting PP2A needs to be explored in the future. In particular, determining how NCTD affects Smad expression and phosphorylation by inhibiting PP2A should be evaluated.

### Conclusions

In our study, the role of NCTD in preserving the tubular epithelial phenotype was confirmed in a rat model of tubulointerstitial fibrosis induced by UUO. Consistent with the *in vivo* results, significant changes in the cell phenotype were observed in HK-2 cells induced by TGF-β_1_, including downregulation of E-cadherin expression and increased α-SMA expression. We infer that treatment with NCTD may attenuate renal fibrosis and inhibit tubular EMT. The mechanisms behind the effects of NCTD on tubular EMT appear to be related to regulation of the TGF-β_1_/Smad pathway and expression of Snail 1. The data obtained in this study identify a new therapeutic target of NCTD for inhibiting EMT and treating related diseases.
