# Correction: Norcantharidin Inhibits Renal Interstitial Fibrosis by Blocking the Tubular Epithelial-Mesenchymal Transition

**DOI:** 10.1371/annotation/84e623b5-d1a7-47b7-96d7-4f5464b6f4b3

**Published:** 2013-10-08

**Authors:** Ying Li, Yan Sun, Fuyou Liu, Lin Sun, Jun Li, Shaobin Duan, Hong Liu, Youming Peng, Li Xiao, Yuping Liu, Yiyun Xi, Yanhua You, Hua Li, Min Wang, Shuai Wang, Tao Hou

A funding organization and grant were incorrectly omitted from the Funding Statement. The Funding Statement should read: "This study was supported by the National Natural Science Foundation of China (Grant No.81100486), the Hunan Provincial Science and Technology Program(Grant No.2013SK3036), the Natural Science Foundation of Hunan Province of China (Grant No.10JJ2011), and the scientific project of the Research Center of Metabolic Syndrome in Central South University of China (Grant No.DY-2008-02-03). The corresponding author and all the above grand funders played a role in designing and doing the experiment and writing the manuscript." 

